# The American Association of Obstetricians and Gynecologists

**Published:** 1889-11

**Authors:** 


The American Association of Obstetricians and Gynecologists, at its recent annual meeting, held in Cincinnati, O., elected the following officers to serve for the ensuing year:

*President*—Dr. E. E. Montgomery, of Philadelphia.

*Vice-Presidents*—Drs. William H. Myers, of Fort Wayne; Dr. R. L. Banta, of Buffalo.

*Secretary*—Dr. William Warren Potter, of Buffalo.

*Treasurer*—Dr. X. O. Werder, of Pittsburgh.

*Executive Council*—Drs. A. Vander Veer, of Albany; Clinton Cushing, of San Francisco; Chas. A. L. Reed, of Cincinnati; William H. Wathen, of Louisville; Hampton E. Hill, of Saco, Me.

The third annual meeting will be held in Philadelphia, beginning on the *third Tuesday* in September, 1890.

